# Association between peri-implantitis and systemic inflammation: a systematic review

**DOI:** 10.3389/fimmu.2023.1235155

**Published:** 2023-08-24

**Authors:** Yumeng Yan, Marco Orlandi, Jeanie Suvan, Simon Harden, Jacqueline Smith, Francesco D’Aiuto

**Affiliations:** ^1^ Periodontology Unit, University College London (UCL) Eastman Dental Institute, University College London (UCL), London, United Kingdom; ^2^ Department of Statistical Science, University College London (UCL) Eastman Dental Institute, University College London (UCL), London, United Kingdom; ^3^ Library Services, University College London (UCL), London, United Kingdom

**Keywords:** systemic inflammation, inflammation, C-reactive protein, peri-implantitis, biomarker, immunity

## Abstract

**Background:**

Peri-implantitis is an infectious/inflammatory disease with similar clinical and radiographic features to periodontitis. Overwhelming evidence confirmed that periodontitis causes elevations in systemic inflammatory mediators; this is unclear for peri-implantitis. Hence, this study aimed to appraise all available evidence linking peri-implantitis with systemic inflammation.

**Methods:**

A systematic review was completed according to Preferred Reporting Items for Systematic Reviews and Meta-Analyses (PRISMA) guidelines. Eight electronic databases (Cochrane Central Register of Controlled Trials, MEDLINE, EMBASE, Web of Science, Dentistry & Oral Sciences Source, Scopus, LILACS, and China Online), ClinicalTrials.gov, WHO International Clinical Trials Registry Platform (ICTRP), and gray literature were searched up to February 9, 2023. Human studies of randomized controlled trials, non-randomized intervention studies, cohort studies, case–control, and cross-sectional studies were eligible for inclusion. Quantitative analyses were performed using random effects models.

**Results:**

A total of 27 full-text articles were retrieved, and 11 clinical studies were included in the final analyses. All evidence gathered demonstrated a consistent association between peri-implantitis and systemic inflammation. Patients with peri-implantitis exhibited higher levels of serum C-reactive protein (CRP) (standard mean difference (SMD): 4.68, 98.7% CI: 2.12 to 7.25), interleukin-6 (IL-6) (weighted mean difference (WMD): 6.27 pg/mL, 0% CI: 5.01 to 7.54), and white blood cell counts (WMD: 1.16 * 10^3^/μL, 0% CI: 0.61 to 1.70) when compared to participants without peri-implantitis.

**Conclusion:**

Peri-implantitis is associated with higher systemic inflammation as assessed by serum CRP, IL-6, and white blood cell counts. Further research is needed to clarify the nature of this association.

**Systematic review registration:**

https://www.crd.york.ac.uk/prospero/display_record.php?RecordID=246837, identifier CRD42021246837.

## Introduction

Over the last three decades, dental implants have been proven to be an effective treatment for replacing teeth. A study reported that the survival rates of dental implants exceeded 85% after 25 years of follow-up (1), but they are not free from complications (2). The prevalence of peri-implant diseases is increasing. Evidence proved that the development and progression of peri-implant disease can result in the eventual loss of dental implants (3), while the definition of peri-implantitis continues to be a controversial issue due to the various case definitions of the disease reported (4). The 2017 International Classification of Periodontal and Peri-Implant Diseases and Conditions defined peri-implantitis as the presence of bleeding and/or suppuration on gentle probing combined with more than 6-mm probing depths and bone loss ≥3 mm (5). Peri-implant diseases display some unique features; the amount of surface area and inflammatory cell composition differentiate peri-implant lesions from periodontal pockets (4, 5).

However, periodontitis not only is linked to local inflammation but also triggers a systemic host response (6). This is usually assessed by serological biomarkers including C-reactive protein (CRP) and interleukin (IL)-6 (7). A raised systemic inflammatory state has been advocated as a potential mechanism linking periodontitis to a variety of systemic diseases including cardiovascular and metabolic diseases (8, 9). Some evidence also suggests that peri-implantitis with its local pathogen and inflammatory burden could be an unrecognized trigger of systemic inflammation (10). With the increasing use of dental implants including in patients with existing co-morbidities, it is therefore unclear what systemic impact peri-implant disease/infection might have.

We therefore aimed to conduct a systematic appraisal of all published evidence evaluating the association between peri-implantitis and its treatment and systemic inflammation.

## Materials and methods

A systematic review was conducted according to the Cochrane Handbook and Preferred Reporting Items for Systematic Reviews and Meta-Analyses (PRISMA) guidelines (11), and the protocol was registered in the PROSPERO register (reference no: CRD42021246837). The focused research questions were “Is there an association between peri-implantitis and systemic inflammation in adults?” and “Is there an association between the treatments of peri-implantitis and systemic inflammation in adults?”, using the PECOS/PICOS.

### PECOS

**P (Patients):** Adult population. Studies that recruited participants who were under 18 years old, pregnant, lactating, or taking antibiotics for purposes other than an intervention to treat peri-implantitis were excluded.

**E (Exposure):** Diagnosis of peri-implantitis according to respective definitions in their studies.

**C (Comparison):** Individuals without peri-implantitis.

**O (Outcome):** Systemic inflammation assessed by

1. serum CRP level (primary), and
2. any other biomarkers (such as white blood cell (WBC) counts, interleukin-6, interleukin-10, necrosis factor-α, interleukin-1β, neutrophils, hemoglobin (Hb), platelets (PLT), and lymphocytes) in peripheral blood (secondary).

**S (Study designs):** Human studies including randomized controlled trials, non-randomized intervention studies, cohort studies, case–control, and cross-sectional studies. However, case reports and series, reviews, and animal studies were excluded.

### PICOS

**P (Patients):** Adult population. Studies that recruited participants who were under 18 years old, pregnant, lactating, or taking antibiotics for purposes other than an intervention to treat peri-implantitis were excluded.

**I (intervention):** Peri-implantitis treatments including non-surgical or surgical implant decontamination with or without other adjunctive therapies.

**C (Comparison):** No treatment or control intervention (supra-mucosal cleaning, oral hygiene instructions, and/or community dental treatment).

**O (Outcome):** Systemic inflammation assessed by

1. serum CRP level reduction (primary) at 3 and 6 months of follow-up, and
2. any other biomarkers (such as white blood cell counts, interleukin-6, interleukin-10, necrosis factor-α, interleukin-1β, neutrophils, hemoglobin, platelets, and lymphocytes) in peripheral blood (secondary) at 3 and 6 months of follow-up.

**S (Study designs):** Human studies including randomized controlled trials.

### Information sources and searches

Broad and inclusive electronic search strategies were designed and conducted to include citations until February 9, 2023. The following electronic databases were searched without language limitation using medical subject headings and free-text terms: Cochrane Central Register of Controlled Trials (CENTRAL), MEDLINE, EMBASE, Web of Science, Dentistry & Oral Sciences Source, Scopus, LILACS, and China Online (example-Medline search **Supplementary Table 1**). Further, SIGLE was used to search for gray literature. The following journals were searched by hand since 2002: *Journal of Periodontology*, *Journal of Clinical Periodontology*, and *Clinical Oral Implants Research*. Registered studies were searched from ClinicalTrials.gov and WHO International Clinical Trials Registry Platform (ICTRP). Resulting hits from the application of the search strategies were imported into a reference manager software (EndNote, version 20).

### Study selection and data extraction

The titles and abstracts (when available) identified through the search were screened independently by two reviewers (YY and MO) based on the inclusion and exclusion criteria. Any disagreement was resolved by discussion. Full reports were assessed for studies which there was insufficient information in the title and abstract to make a clear decision. The full reports were assessed independently, in duplicate, by the same reviewers to establish eligibility for inclusion. If necessary, a third reviewer was consulted (FD). Data extracted and collated in evidence tables included study characteristics, population, exposure, intervention, outcomes, and author conclusions.

### Quality assessment of selected studies

Descriptive analysis was performed to determine the quality of data, checking further for study variations, in terms of study characteristics, and results and assessing suitability for inclusion in meta-analysis. Quality assessment of included studies was undertaken independently by two reviewers (YY and MO). Quality assessment and risk of bias in observational studies, randomized controlled trials, and non-randomized studies of interventions were determined using the Newcastle-Ottawa Scale (NOS) (12), the revised Cochrane tool (RoB 2) (13), and the ROBINS-I tool (14), respectively.

### Analyses

Qualitative analyses for all included studies were completed and reported in evidence tables. Further quantitative analyses were performed on all available evidence retrieved using Stata/MP 17.0 (StataCorp, College Station, TX, USA). Weighted mean differences (WMDs) with a 95% confidence interval (CI) were calculated for parameters reported and assessed using similar methods, while for those assessed and reported with different methods/units, standard mean difference (SMD) was applied.

The heterogeneity of the data was assessed using the c2-based Q-statistic method and considered significant if p < 0.05 and quantified with the I-squared statistic. Pooled estimates were calculated using random effects models when at least two studies with the data were available. Potential inter-study heterogeneity was considered to adopt more conservative analyses. The pooled effect was considered significant if p < 0.05. Publication bias was examined using a funnel plot and Egger’s test (15). Sensitivity analyses were defined *a priori* to understand the influence of individual studies on the aggregate estimates. Meta-analyses of different inflammatory biomarkers/outcomes were evaluated based on the Grading of Recommendations Assessment, Development, and Evaluations (GRADE) approach (16).

## Results

### Selection and characteristics of included studies

**Figure 1:**
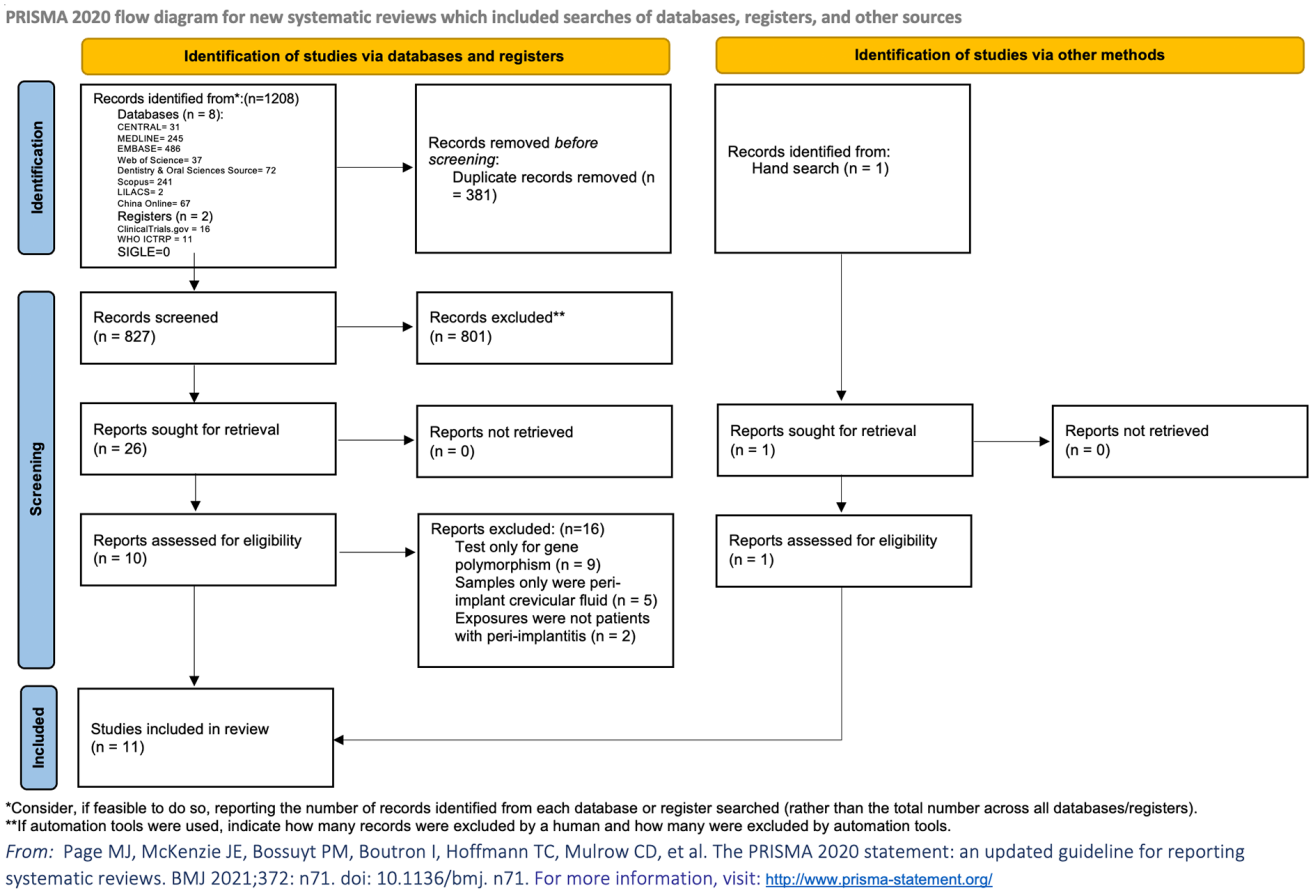
PRISMA flowchart. Flowchart of this study shows the process that we followed to identify and select studies. The original databases and registers retrieved 1,208 records. A total of 827 citations remained after removal of duplicates; 26 articles were retrieved for full-text assessment databases and registers. Another record was identified by hand search. A total of 11 articles were eligible after the application of inclusion and exclusion criteria.

The electronic search identified 1,208 articles of potential relevance. After the removal of duplicates, 827 citations remained. After title and abstract screening, 26 articles were retrieved for full-text assessment from databases and registers, and one record was identified by hand searching. Finally, a total of 11 articles were eligible for inclusion in the review, among which seven were case–control studies (17–23), and four were cross-sectional studies (24–27) (**Table 1**; **Figure 1**). Five studies (17, 19, 22, 25, 26) compared participants with healthy implants. Another four studies only mentioned that participants from comparison groups were with healthy oral conditions (18, 20, 21, 24) and did not specify whether dental implants existed or not. Two studies (23, 26) divided the comparison groups into two groups, which were participants with healthy dental implants, or without dental implants but with healthy periodontium. Two studies defined the inclusion criteria in their studies as the presence of at least one implant diagnosed with peri-implantitis (17, 27), and another study divided peri-implantitis patients into different groups based on dental implant numbers and severity of peri-implantitis (24). Other included studies did not clearly illustrate the number of implants that were diagnosed with peri-implantitis. No randomized controlled trials were retrieved. A total of 32 blood parameters were measured to investigate the association between peri-implantitis and systemic inflammation (**Supplementary Table 2**). After the assessment of available data, one study was not eligible to be included in the meta-analysis due to a lack of standard deviation (20), and another article presented the data only as medians (25). Finally, nine articles were included in the quantitative analysis.

**Table 1:** Evidence table for included studies.

Participants’ characteristics and study design of the 11 studies included in the meta-analysis					
Author, year of publication, and country	Study design	Definition of peri-implantitis	Ages a. Peri-implantitis b. Controls	n, peri-implantitis/controls	Serum biomarkers (parameters) measured
Ustaoğlu and Erdal, 2020, Turkey (19)	Case–control	The presence of bleeding and/or suppuration during gentle probing and radiographic bone levels of at least 3 mm apical of the most coronal fragment of the intra-osseous part of the implant	a = 55 (17.5) b = 50 (18.5)	58/49	Low-density lipoprotein-cholesterol, high-density lipoprotein-cholesterol, triglyceride, total cholesterol, uric acid, creatine, white blood cells, neutrophil, hemoglobin, mean corpuscular volume, platelets, mean platelet volume, platelet crit
Mustafaev et al., 2017, Russia (24)	Cross-sectional	Class III according to classification of S. A. Jovanovic (1990) and N. Spiekermann (1991)	a = 43 (9-57) b = 37 (26-48)	32/25*	Interleukin-1β, interleukin-6, interleukin-10, interleukin-17A, CD401
Khatavkar et al., 2021, India (20)	Case–control	NA	NA	40/40	C-reactive protein
Kour et al., 2020, India (18)	Case–control	NA	NA	60/60	C-reactive protein
Hultin et al., 2002, Sweden (17)	Case–control	Compared to the 1-year intraoral radiographic examination, they had at least one implant showing radiographic marginal bone loss of three or more fixture threads (1.8 mm) mesially or distally	a = 62.8 (7.7) b = 65.1 (6.7)	17/19	C-reactive protein-sensitive, haptoglobin, α1-antitrypsin, red blood cells, hemoglobin, white blood cells, neutrophil, lymphocytes, monocytes, eosinophils, basophils
Wang, 2021, China (22)	Case–control	Gingival crevicular bleeding index >1, periodontal probing depth ≥5 mm, mucosa swelling, with or without suppuration, vertical alveolar bone loss, bleeding on gentle probing	a = 40.5 (6.5) b = 40.6 (7.0)	14/66	C-reactive protein
Wu and Gao, 2019, China (23)	Case–control	Gingival crevicular bleeding index >1, periodontal probing depth ≥5 mm, and the implants are not loose nor any sinus around the implants	NA	17/113^†^	C-reactive protein
Khichy et al., 2021, India (21)	Case–control	Hashim D, Cionca N, Combescure C, Mombelli A. The diagnosis of peri-implantitis: A systematic review on the predictive value of bleeding on probing. Clin Oral Implants Res 2018;29 Suppl 16:276-93	a = 41.3 b = 39.6	20/20	C-reactive protein, interleukin-6
Blanco et al., 2021, Span (27)	Cross-sectional	Case definitions of peri-implantitis and periodontitis followed the guidelines of consensus reports of 2017 World Workshop (AAP/EFP) (presence of bleeding on probing [BoP] and/or suppuration, probing pocket depth [PPD] ≥6 mm and/or ≥3 mm of radiographic bone loss)	a = 57.4 (10) b = 57.4 (9.1)	16/31	Total cholesterol, triglycerides, low-density lipoprotein-cholesterol, high-density lipoprotein-cholesterol, total/high-density lipoprotein-cholesterol ratio, low-density lipoprotein-cholesterol/high-density lipoprotein-cholesterol ratio, creatinine, glucose, red blood cells, platelets, white blood cells, lymphocytes, monocytes, neutrophils, eosinophils, basophils, tumor necrosis factor-α, interleukin-10
Wang et al., 2021, USA (26)	Cross-sectional	Case definitions of peri-implantitis and periodontitis followed the guidelines of consensus reports of 2017 World Workshop (AAP/EFP)	NA	54/40	Interleukin-1β, interleukin-6, matrix metalloproteinase-8, osteoprotegerin, tumor necrosis factor-α, high sensitive C-reactive protein, fibrinogen
Ozgur et al., 2023, Turkey (25)	Cross-sectional	Case definitions of peri-implantitis and periodontitis followed the guidelines of consensus reports of 2017 World Workshop (AAP/EFP)	a = 56.4 (7.08) b = 45.2 (12.8)	22/23	C-reactive protein, interleukin-6, soluble ST2

NA, not applicable because not reported.

* Patients were divided into two groups according to severity of the peri-implantitis: first group, 17 patients (53.1%) with moderate-sized peri-implantitis; second group, 15 patients (46.9%) with severe peri-implantitis. Total number of patients with peri-implantitis is 32.

^†^ Control patients were combined with healthy peri-implant (n = 83) and healthy periodontium without implants (n = 30).

### Risk of bias

Risk of bias assessment of observational studies, evaluated using the Newcastle-Ottawa Scale, was based on a) the selection and definition of controls, b) hospital controls rather than community population, c) ambiguous definitions and descriptions of the history of peri-implantitis, and d) non-response rate. The majority of observational studies were considered at low risk of bias (17, 19, 21–27), while only two studies were at high risk of bias (18, 20). The most common sources of bias among included articles were non-response rate and selection of controls (**Supplementary Table 3**). Publication bias analysis could not be conducted due to the limited number of studies retrieved/available. Due to the limited number of included articles and small sample size, the outcomes could be influenced by inconsistency and imprecision (**Supplementary Figure 1**). Sensitive analysis was conducted by omitting one article each time (**Supplementary Figure 2**).

### CRP

**Figure 2:**
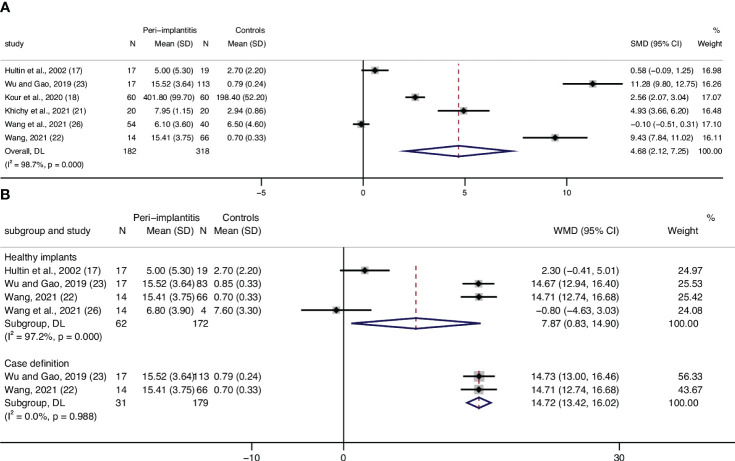
Forest plot for C-reactive protein (CRP) between cases (peri-implantitis) and controls (without peri-implantitis). Forest plot for standard mean difference (SMD) and weight mean difference (WMD) (95% confidence interval [CI]). Random effects model was used. The squares represent the relative effect of studies; the diamond represents the overall effect of all the studies. **(A)** Peri-implantitis patients (n = 182) showed higher serum CRP levels than controls (n = 318), SMD of 4.68 [2.12, 7.25], with high level of heterogeneity (I^2^ = 98.4%). **(B)** Healthy implants: subgroup analysis of patients in control groups of participants with healthy implants. Forest plots indicated higher serum CRP levels in patients with peri-implantitis (n = 62) than the controls (n = 172), WMD of 7.87 mg/L [0.83, 14.9]. **(B)** Case definition: subgroup analysis of the studies that defined peri-implantitis as gingival crevicular bleeding index >1 and periodontal probing depth ≥5 mm. Results showed significantly higher serum CRP levels in peri-implantitis patients than those without peri-implantitis.

Eight articles assessed CRP levels, while one of the studies lacked standard deviation, and one study only presented CRP as medians in their article. Meta-analysis of six articles confirmed a statistically significant higher level of CRP in patients with peri-implantitis (n = 182) compared to patients without peri-implantitis (n = 318); SMD of 4.68 [2.12, 7.25], with high level of heterogeneity (I^2 =^ 98.7%), was detected, which means higher levels of CRP in patients with peri-implantitis (**Supplementary Table 4**; **Figure 2A**).

### Subgroup analysis for control groups of patients with healthy implants

Analyses were conducted for the studies that specified that their controls were patients with healthy implants, and results showed WMD of 7.87 mg/L [0.83,14.90], with a heterogeneity of 97.2%, which shows mean CPR levels in patients with peri-implantitis of 7.87 mg/L higher than that of patients without peri-implantitis (**Figure 2B**, Healthy implants).

### Subgroup analysis based on the case definition of peri-implantitis

Subgroup analysis based on the case definitions was also performed. Included studies have different definitions according to probing depth, bone loss, bleeding on probing, or swelling/suppuration. Two studies (22, 23), which defined peri-implantitis as gingival crevicular bleeding index >1 and periodontal probing depth ≥5 mm, revealed a significantly lower level of CRP in patients without peri-implantitis compared to patients with peri-implantitis (**Table 1**; **Figure 2B**, Case definition). Two studies (25, 26) defined peri-implantitis following the guidelines of consensus reports of the 2017 World Workshop. No significant difference in CRP levels was discovered between patients with peri-implantitis and without.

### WBC

**Figure 3:**
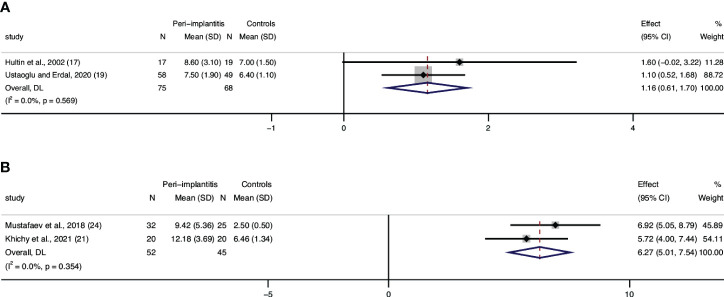
Forest plot for white blood cell (WBC) count interleukin-6 (IL-6) between cases (peri-implantitis) and controls (without peri-implantitis). Forest plot for mean difference (95% confidence interval [CI]). Random effects model was used for weight mean difference (WMD). The squares represent the relative effect of studies; the diamond represents the overall effect of all the studies. Higher levels of WBC **(A)** and IL-6 **(B)** in peri-implantitis groups compared with controls were revealed.

Three articles (17, 19, 27) assessed WBC levels, and data from two articles were eligible to be included in a meta-analysis. Results from the meta-analysis confirmed statistically significant higher leucocyte counts in patients with peri-implantitis (n = 75) compared to those without peri-implantitis (n = 68), with a low level of heterogeneity (0%) (**Figure 3A**). Although results from Blanco et al. cannot be included in quantitative analysis, they also reported a significantly lower level of WBC in patients without peri-implantitis than in peri-implantitis patients (27).

### Cytokines

Two studies that assessed serum IL-6 and were included in the meta-analysis showed significant differences between patients with peri-implantitis and without (**Figure 3B**). Regarding IL-1β, a study by Mustafaev et al. confirmed a significantly lower level of serum IL-1β in patients with healthy periodontium/implants than in patients with peri-implantitis (24). Blanco also announced that patients with peri-implantitis had a significantly higher level of TNF-α than patients with healthy implants (27).

### Other biomarkers

Meta-analyses for neutrophils, hemoglobin, platelets, and lymphocytes were also conducted, while no significant differences were observed in these mediators for patients with peri-implantitis versus those without peri-implantitis (**Supplementary Figure 3**).

## Discussion

This systematic review and meta-analysis indicates that peri-implantitis is not only associated with local mucosal inflammation but rather accompanied by a systemic host response. It is the first critical appraisal of the limited but consistent evidence suggesting that peri-implantitis could have systemic implications (i.e., patients with existing co-morbidities linked to systemic inflammation).

A recent review discussed the relationship between peri-implantitis and systemic diseases pointing at a possible role of body response to peri-implantitis, but due to the limited evidence, authors urged caution to avoid overinterpretation of the data available (10). Hultin et al. first reported a not statistically significant trend of higher serum CRP levels in patients with peri-implantitis (17). More recently, reports confirmed that higher serum CRP levels are associated with peri-implantitis (18, 20–23, 26). Moreover, two studies revealed that serum CRP significantly decreased after treating peri-implantitis with local metronidazole gel (22, 23). The main limitation of the evidence reported so far, however, is that most of the clinical trials performed were not primarily designed to test the impact of peri-implantitis on systemic inflammation. CRP has been used as a marker of systemic inflammation in these studies, and overwhelming evidence justifies this choice (28). Although CRP can be generated in reconstituted human gingival epithelia (29), which may explain the systemic inflammation induced by pathological periodontal tissues, it can also be released directly by the liver as part of the common acute phase response (30). Systemic inflammatory changes could impact the onset and progression of other systemic disorders (i.e., cardiometabolic diseases), and recent evidence points toward specific immune alterations (trained myelopoiesis) as potential common mechanisms (31, 32). It is plausible that peri-implantitis as periodontitis could represent an enhanced inflammatory stimulus with systemic implications, and we could speculate that the mechanism of CRP release due to periodontal inflammation is similar to that observed in patients with peri-implantitis.

There is plenty of evidence about the role of early inflammatory markers during the development of the host inflammatory response (i.e., cytokines) (33). This review included all reported inflammatory biomarkers rather than CRP, which have been measured in patients with peri-implant disease. As a multifunctional cytokine, IL-6 is secreted by several types of cells and is active in both innate and adaptive immune responses (34). Studies have reported that periodontitis is accompanied by increased production of proinflammatory cytokines (35, 36). Moreover, it has been demonstrated that proinflammatory cytokines in peri-implantitis crevicular fluid are increased (37) and anti-inflammatory cytokines are decreased (38). In this review, two studies (21, 24) also demonstrated higher concentrations of IL-6 in patients with peri-implantitis than without. Among other pro-inflammatory markers, IL-1β, TNF-α, and IL-17 have been extensively studied in the context of the generation of local and systemic inflammation and in particular relevant to periodontal inflammation and later tissue destruction (39, 40). There is currently evidence linking peri-implantitis to increased local production of TNF-α and IL-17 (41, 42). Evidence on the increased levels systemically of these markers in patients with dental implants and related diseases is limited. Higher serum concentrations of IL-1β and TNF-α were detected in patients with peri-implantitis compared to those without peri-implantitis (24, 27). Inconclusive evidence exists on the levels of IL-10 in patients with peri-implantitis (24, 27). Hematological changes were reported to be associated with peri-implantitis. In an experimental animal study, substantial differences in WBC, Hb, red blood cells (RBC), mean corpuscular hemoglobin (MCH), mean corpuscular hemoglobin concentration (MCHC), PLT, and mean corpuscular volume (MCV) were observed subsequent to the onset of experimental peri-implantitis (43). This review confirmed that peri-implantitis is associated with higher WBC count (a crude measure of systemic inflammation). These results are consistent with the evidence retrieved in patients with periodontitis (44), which reflect the inflammatory response of human body to peri-implantitis.

**Figure 4:**
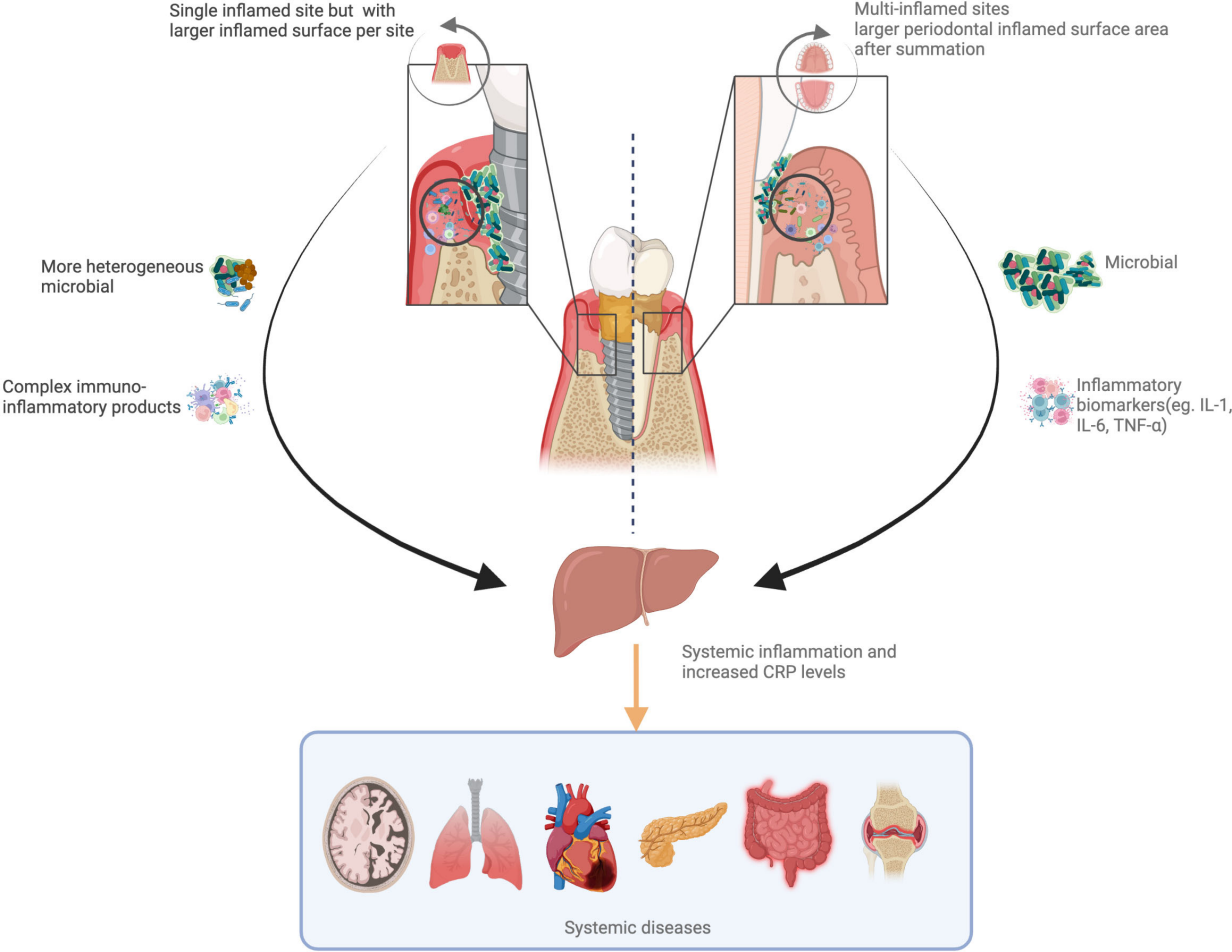
Hypothetical pathways linking peri-implantitis/periodontitis to systemic inflammation. Periodontitis can increase the risk of systemic inflammation and boost the C-reactive protein (CRP) levels with full mouth inflammation. Peri-implantitis always occur as single site, while a more heterogeneous bacterial flora and complex immune-inflammatory products could induce greater systemic inflammation. These two pathways add to systemic health risks.

The exact mechanism behind the systemic impact of peri-implantitis remains unclear. Blanco et al. suggested that peri-implantitis subgingival pathogens induce a local inflammatory response followed by local exaggerated production of pro-inflammatory cytokines and prostaglandins (27). Histological evidence confirmed the greater inflammatory infiltrate within the peri-implant mucosa when peri-implantitis is diagnosed (45). Thus, it is reasonable to hypothesize that peri-implantitis could induce a systemic inflammatory response in a similar fashion to periodontitis (46) (**Figure 4**). A recent report confirmed indeed that when contrasting the host response of patients with periodontitis and peri-implantitis, the former represented a greater stimulus/trigger (47). This could be explained by the disproportionate number of inflamed sites often identified in patients with periodontitis as opposed to most reports describing the host response in patients with peri-implantitis referring to the single-digit number of implants affected. The main difference between these two conditions would therefore be the relative amount of inflamed surface area. Indeed, it is accepted that the periodontal inflamed surface area could extend up to 40 cm^2^ (48), while it is unclear how peri-implant inflamed mucosa would differ or be similar. The lack of reliable measures of the amount of gingival inflammation/cellular infiltrate within the peri-implant tissue could influence an accurate evaluation of the local and systemic impact of peri-implantitis.

Finally, there is a lack of randomized controlled trials on the topic. This would represent the highest level of evidence and address the issue of causality when studying the association between peri-implantitis and systemic inflammation/host response. We urge researcher colleagues therefore to focus on designing adequately powered clinical trials to address this question and ascertain the potential systemic implications of peri-implant diseases.

We should acknowledge some limitations of this review such as the high risk of bias of some trials included in our analysis and the differences in the definition of peri-implantitis as well as in the laboratory assessment of CRP. We strongly believe that these factors might have contributed to the high level of heterogeneity detected. Caution in interpreting these data conservatively should be exerted, and further and better-designed observational studies should be reported. In contrast, our systematic review methodology was based on a preregistered protocol, with a strict methodology and detailed process, including all possible confounding factors when assessing the available evidence, and could give confidence when interpreting the quantitative analyses.

In conclusion, our analysis supports an association between peri-implantitis and systemic inflammation as assessed by serum CRP levels. Although the mechanisms behind this association are still unclear, our findings are relevant when exploring potential systemic implications of overlooked sources of systemic inflammation from the oral cavity (i.e., peri-implantitis), especially in patients with other co-morbidities.

## Data availability statement

The original contributions presented in the study are included in the article/**Supplementary Material**. Further inquiries can be directed to the corresponding author.

## Author contributions

YY contributed to the conception, design, data acquisition, and interpretation, and drafted and critically revised the manuscript. MO contributed to the conception and data acquisition and critically revised the manuscript. JSu contributed to the data interpretation, and drafted and critically revised the manuscript. SH contributed to the statistical analyses. JSm contributed to the search strategy. FD’A contributed to the conception, design, and data interpretation, and drafted and critically revised the manuscript. All authors contributed to the article and approved the submitted version.
