# Supplementary material for: Association between peri-implantitis and systemic inflammation: a systematic review
Source: Front Immunol. 2023 Aug 24;14:1235155. doi: 10.3389/fimmu.2023.1235155 (PMC10484704; doi:10.3389/fimmu.2023.1235155)
Supplement: Supplementary file 1 [file DataSheet_1.pdf]

Association Between Peri-implantitis and Systemic inflammation: A Systematic Review

RUNNING TITLE: Systemic inflammation and Peri-implantitis

Yumeng Yan[1], Marco Orlandi[1], Jeanie Suvan[1], Simon Harden[2], Jacqueline Smith[3], Francesco D'Aiuto[1]

Affiliations:
[1] Periodontology Unit, UCL Eastman Dental Institute, UCL
[2] Department of Statistical Science, UCL Eastman Dental Institute, UCL
[3] UCL Library Services.

Corresponding Author:

Prof Francesco D'Aiuto
Periodontology Unit
UCL Eastman Dental Institute
21 University St, Bloomsbury,
London WC1E 6DE
f.daiuto@ucl.ac.uk, ORCID iD: 0000-0001-8654-935X

List of figures

Supplemental Figures 1: Sensitivity analysis for serum CRP
Supplemental Figures 2: GRADEpro assessment tool
Supplemental Figures 3: Forest plots for neutrophils, lymphocytes, platelets, haemoglobin of cases(peri-implantitis) versus healthy controls

Supplemental Figures 1: Sensitivity analysis for serum CRP

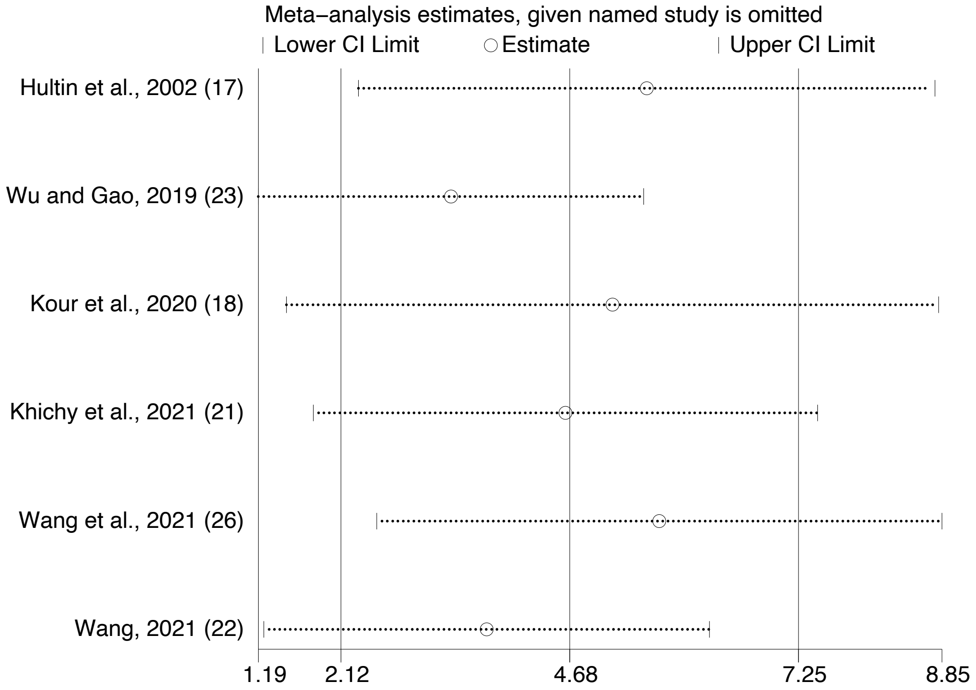

Sensitivity analysis for serum CRP by omitting one study each time

Supplemental Figures 2: GRADEpro assessment tool

Question: Serum CRP level in Patients with peri-implantitis compared to patients without peri-implantitis
Setting:
Bibliography:

Certainty assessment							№ of patients		Effect		Certainty	Importance
№ of studies	Study design	Risk of bias	Inconsistency	Indirectness	Imprecision	Other considerations	Patients with peri-implantitis	patients without peri-implantitis	Relative (95% CI)	Absolute (95% CI)		
**Serum CRP level**												
6	observational studies	not serious	serious[a]	not serious	serious[b]	all plausible residual confounding would reduce the demonstrated effect dose response gradient	182 cases 318 controls		**SMD 4.68** (2.12 to 7.25)	-	⊕⊕◯◯ Low	
							-	0.0%		**-- per 1,000** (from -- to --)		

CI: confidence interval

Explanations

a. heterogeneity is high (98.7%).
b. limited number of included articles and small sample size

The outcomes were influenced by inconsistency and imprecision.

Supplemental Figures 3
Forest plots for neutrophils, lymphocytes, platelets, haemoglobin of cases(peri-implantitis) versus healthy controls

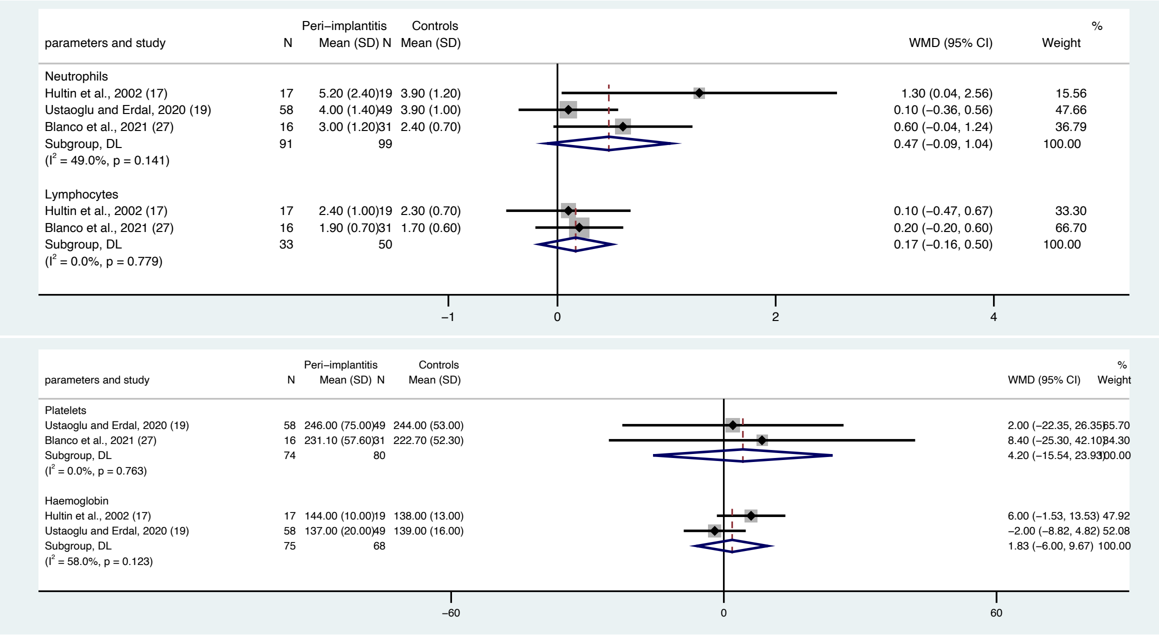

Supplemental Figure 3: Forest plot for neutrophils, lymphocytes, platelets, haemoglobin of peri-implantitis versus healthy controls. Forest plot for weight mean difference (95% confidence interval [CI]), random effects model was used. The squares represent the relative effect of studies, the diamond represents the overall effect of all the studies.

List of tables

Supplemental Table 1: Search strategy for MEDLINE via OVID
Supplemental Table 2: Parameters assessed in include articles
Supplemental Table 3: Bias assessment for observational studies Newcastle Ottava scale
Supplemental Table 4: Summary table of included studies - meta-analyses outcomes for all the parameters

Supplemental Table 1: Search strategy for MEDLINE via OVID

**Supplemental Table 1: Search strategy for MEDLINE via OVID**
1. exp Peri-implantitis/
2. exp Dental implants/
3. exp Dental implantation/
4. peri-implant*.mp.
5. periimplant*.mp.
6. (inflammation adj1 implan*).mp.

7. or/1-6
8. exp Dental Scaling/
9. exp Air Abrasion, Dental/
10. perio-flow.mp.
11. perioflow.mp.
12. airflow.mp.
13. air-flow.mp.
14. "hand instrumen*".mp.
15. "ultrasonic scal*".mp.
16. "mucoperiosteal fla*".mp.
17. mucoperiosteal.mp.
18. photodynamic.mp.
19. "photodynamic therap*".mp.
20. (scal* adj4 polish*).mp.
21. ((implant adj6 scal*) or (implants adj6 scal*)).mp.
22. (gingivectomy or gingivoplasty or "subgingival curettage").mp.
23. exp Surgical flaps/
24. surgical fla*.mp.
25. PDT.mp.
26. exp Chlorhexidine/
27. exp Anti-Bacterial Agents/
28. exp Tetracycline/
29. (antibiotic* or anti-biotic* or antibacterial* or anti-bacterial*).mp.
30. (tetracycline* or doxycycline* or minocycline* or roxitomycin* or moxifloxacin* or ciprofloxacin* or metronidazole*).mp.
31. "non-surgical therap*".mp.
32. "surgical therap*".mp.
33. treatmen*.mp.
34. or/8-33
35. (circulate* or "peripheral blood" or wholeblood or whole-blood or "whole blood" or blood or serum).mp.
36. exp C-reactive Protein/
37. exp Leukocyte Count/
38. exp Interleukin/
39. exp erythrocyte sedimentation Rate/
40. CRP.mp.
41. "acute phase protein".mp.
42. "white cell coun*".mp.
43. ESR.mp.
44. biomarker*.mp.
45. "inflammatory marker*".mp.
46. "marker* of inflammat*".mp.

47. exp neutrophil/
48. exp lymphocyte/
49. exp Platelet to lymphocyte ratio/
50. exp Lymphocyte to monocyte ratio/
51. exp Monocyte/
52. exp Platelet count/
53. exp Hemoglobin/
54. exp Cytokines/
55. exp Neutrophil to lymphocyte ratio/
56. LYM.mp.
57. PLR.mp.
58. NEU.mp.
59. LMR.mp.
60. MONO.mp.
61. PLT.mp.
62. HGB.mp.
63. or/36-61
64. 35 and 63
65. 7 and 34 and 64
66. 7 and 64

Supplemental Table 2: Parameters assessed in included articles

Parameter	Study
C-reactive protein	Hultin et al., 2002, Khatavkar et al., 2021, Kour et al., 2020, Khichy et al., 2021, Wu and Gao, 2019, Wang et al., 2021, Wang, 2021, Ozgur et al., 2023
IL-1β	Mustafaev et al., 2017, Wang et al., 2021
IL-6	Mustafaev et al., 2017, Khichy et al., 2021, Wang et al., 2021, Ozgur et al., 2023
IL-10	Mustafaev et al., 2017, Blanco et al., 2021
IL-17A	Mustafaev et al., 2017
CD 401	Mustafaev et al., 2017
TNF-α	Blanco et al., 2021, Wang et al., 2021
HDL†	Ustaoğlu and Erdal, 2020, Blanco et al., 2021
LDL‡	Ustaoğlu and Erdal, 2020, Blanco et al., 2021
Total cholesterol	Ustaoğlu and Erdal, 2020, Blanco et al., 2021
Total/HDL cholesterol ratio	Blanco et al., 2021
LDL/HDL cholesterol ratio	Blanco et al., 2021
Triglyceride	Ustaoğlu and Erdal, 2020, Blanco et al., 2021
Vitamin D	Ustaoğlu and Erdal, 2020
White blood cell	Ustaoğlu and Erdal, 2020, Blanco et al., 2021b, Hultin et al., 2002
Lymphocyte	Blanco et al., 2021, Hultin et al., 2002
Monocyte	Blanco et al., 2021, Hultin et al., 2002
Eosinophils	Blanco et al., 2021, Hultin et al., 2002
Basophils	Blanco et al., 2021, Hultin et al., 2002
Hemoglobin	Ustaoğlu and Erdal, 2020, Hultin et al., 2002
Haptoglobin	Hultin et al., 2002
Mean corpuscular volume	Ustaoğlu and Erdal, 2020
Mean platelet volume	Ustaoğlu and Erdal, 2020
Plateletcrit	Ustaoğlu and Erdal, 2020
Glucose	Blanco et al., 2021
Platelet counts	Ustaoğlu and Erdal, 2020, Blanco et al., 2021
Red blood cell	Blanco et al., 2021, Hultin et al., 2002
α_1-antitrypsin in plasma	Hultin et al., 2002
Soluble ST2	Ozgur et al., 2023
Matrix metalloproteinase-8	Wang et al., 2021
Osteoprotegerin	Wang et al., 2021
Fibrinogen	Wang et al., 2021

† : HDL: high-density lipoprotein cholesterol
‡ : LDL: low-density lipoprotein cholesterol

Supplemental Table 3: Bias assessment for observational studies Newcastle Ottava scale

Articles	Selection				Comparability	Exposure			Scores
	adequate definition of cases	representativeness of the cases	selection of controls	definition of controls	control for important factor	ascertainment of exposure	ascertainment of exposure	non-response rate	
Hultin et al, 2002,	*	*		*	**	*	*		7
Mustafaev et al, 2017	*	*	*	*	*	*	*		7
Peng and ChengZhi, 2019	*	*		*	**	*	*		7
Ustaoğlu and Erdal, 2020	*	*		*	**	*	*		7
Kour et al, 2020					*	*	*		3
Khatavkar et al, 2021					*	*	*		3
Wang, 2021	*	*		*	**	*	*		7
Khichy et al., 2021	*	*		*	**	*	*		7
Blanco et al, 2021	*	*		*	**	*	*		7
Wang et al., 2021	*	*		*	**	*	*		7
Ozgur et al, 2023	*	*		*	**	*	*		7

Supplemental Table 4: Summary table of included studies - meta-analyses outcomes for all the parameter

**Parameter**	**No. of studies**	**Sample size (peri-implantitis)**	**Sample size (controls)**	**SMD/ WMD**	**95% CI**	**I^2**
c-reactive protein	6	182	318	4.68	**2.12, 7.25**	98.7%
Interleukin-6	2	52	45	6.27	**5.01, 7.54**	0%
white blood cell	2	75	68	1.16	**0.61, 1.70**	0%
neutrophils	3	91	99	0.47	-0.09, 1.04	49%
hemoglobin	2	75	68	1.83	-6.00, 9.67	58%
platelets	2	74	80	4.2	-15.54, 23.93	0%
lymphocytes	2	33	50	0.17	-0.16, 0.50	0%
